# Characterization of Goat Production Systems in the Northern Dry Forest of Peru Using a Multivariate Analysis

**DOI:** 10.3390/ani15040567

**Published:** 2025-02-16

**Authors:** Victor Temoche, Irene Acosta, Pablo Gonzales, David Godoy Padilla, Omar Jibája, Juancarlos Cruz, Flor-Anita Corredor

**Affiliations:** 1 Estación Experimental Los Cedros, Instituto Nacional de Innovación Agraria (INIA), Los Cedros, Corrales, Tumbes 24500, Peru; 2 Departamento Académico de Medicina Veterinaria y Zootecnia, Facultad de Ciencias Agrarias, Universidad Nacional de Tumbes (Untumbes), Los Cedros, Corrales, Tumbes 24500, Peru; ojibajac@untumbes.edu.pe; 3 Estación Experimental Agraria Vista Florida, Instituto Nacional de Innovación Agraria (INIA) Chiclayo, Chiclayo 14006, Peru; ireneacosta9125@gmail.com; 4 Estación Experimental Agraria El Chira, Instituto Nacional de Innovación Agraria (INIA), Marcavelica, Sullana 20120, Peru; pgonzales@inia.gob.pe; 5 Escuela de Posgrado, Unidad de Posgrado de la Facultad de Zootecnia, Universidad Nacional Agraria La Molina, La Molina, Lima l2791, Peru; davgodoy1@gmail.com; 6 Estación Experimental La Molina, Dirección de Supervisión y Monitoreo en las Estaciones Experimentales Agrarias, Instituto Nacional de Innovación Agraria (INIA), La Molina, Lima l2791, Peru; jcruz@inia.gob.pe (J.C.); acorredor117@gmail.com (F.-A.C.)

**Keywords:** goat production systems, multivariate analysis, dry forest, livestock typology, northern Peru

## Abstract

Goat production in the dry forest of northern Peru is essential for rural livelihoods but remains poorly characterized regarding its productivity and sustainability. This study used multivariate techniques—a multiple correspondence analysis (MCA), principal component analysis (PCA), factor analysis of mixed data (FAMD), and hierarchical cluster analysis (HCA)—to analyze data from 284 producers in Tumbes, Piura, and Lambayeque. Surveys captured 48 variables (41 qualitative, seven quantitative) on productivity, socioeconomics, and management. The MCA explained 22.07% of the variability in two dimensions, while the PCA accounted for 63.9%, focusing on productivity and diversification. The FAMD integrated these variables, explaining 51.12% of variability across five dimensions, emphasizing socioeconomic and management differences. The HCA identified three clusters: cluster 1 featured intensive systems with advanced management and commercial focus, cluster 2 included extensive systems limited by water scarcity, and cluster 3 reflected semi-intensive systems with irrigation and diversified production. These findings provide a detailed understanding of goat systems in northern Peru, identifying opportunities to improve resource use and tailor strategies to enhance sustainability. The multivariate analysis proved effective in capturing the complexity of these systems, supporting productivity and improving livelihoods in rural areas.

## Simple Summary

Goat farming is vital in rural communities, offering milk, meat, and financial stability, particularly in dry and resource-limited regions. This study examined goat production systems in northern Peru to explore their diversity and performance. Data from 284 farmers in Tumbes, Piura, and Lambayeque revealed two distinct systems: traditional systems, which rely on limited resources and basic practices; and modernized systems, which incorporate irrigation, improved breeding techniques, and better infrastructure. The modernized systems demonstrated higher productivity and stronger market connections, particularly in producing milk, cheese, and goat kids. In contrast, the traditional systems faced challenges such as limited access to water, outdated practices, and toxic plants. These findings emphasize the potential of modern technologies and improved management practices to boost productivity and livelihoods for rural families. Understanding these systems enables policymakers and organizations to create targeted strategies that enhance sustainability, promote innovation, and strengthen food security in northern Peru and similar regions globally.

## 1. Introduction

Goat farming is essential in providing meat, milk, and cheese to vulnerable and low-income populations worldwide [1,2]. Owing to their impressive ability to thrive in harsh environments [3,4], goats are an invaluable asset for rural communities. They enhance food security and serve as a crucial species in meeting the challenges of climate change through sustainable and profitable production methods [5,6,7].

Peru represents a unique case study due to its rich history of goat breeding, which began in the 16th century with the introduction of Spanish breeds. Goats are distributed across most of the country, except in humid tropical regions, with predominantly high populations in the northern areas, arid coastal valleys, and the Andes [8]. In these three geographic areas, extensive goat production systems have been described, emphasizing the use of crop residue stubble and transhumance practices in coastal valleys [9,10], the use of shrubs and grasslands with transhumance practices in Andean valleys [11,12], and the utilization of grasslands in the dry forest of the northern coast [10].

Currently, the goat population in Peru is approximately 1,764,665 [13], with about 28% located along the northern coast, particularly in the dry forest ecosystem, which is recognized for its extended drought periods and high temperatures [14]. The dry forest is distributed mainly in three regions of Peru: Piura, Tumbes, and Lambayeque [15]. This ecosystem is susceptible to different threats, such as deforestation, fragmentation, overgrazing, fire, changes in land use, and drought [16,17]. Goat feeding predominantly consumes the natural grass of dry forests, taking advantage of native vegetation [18]. For this reason, goat farming has been considered responsible for overgrazing. However, a study showed that this activity had a less negative impact on species richness or diversity of the dry forest of Peru and Ecuador [17]. For many years, meat production has been considered the main goat production target in some regions of the Peruvian northern coast [19]. Nevertheless, significant sociological, economic, cultural, technological, and commercial challenges could have influenced the production systems of small goat farmers nowadays.

To date, various studies have been carried out to classify the dairy goat production systems in arid and semi-arid regions of the world, based on characteristics of farms and management practices, type of resource used, and analyses of typologies [20]. For instance, in Brazil, the socioeconomic, productive, and structural diversity of dairy goat production systems in a semi-arid region was characterized, showing diversity among the identified groups (family system of subsistence, both farmers’ consumption and for local trade, with low technology use) but with similar aspects such as the introduction of specialized breeds for milk production, high stocking rates, a strong dependence on external feeds, and few commercialization alternatives [21,22]. In Mexico, goat farming is widely distributed in arid and semi-arid areas under extensive conditions, with little or no use of technology or grasslands or practicing transhumance, and the goats are mostly fed shrubs and forages [23]. There, three subsystems were defined based on the use of land for grazing: free grazing (including the use of crop residues) with heterogeneous herds; grazing with night confinement, where the primary incomes for farmers comes from sales of dairy products; transhumance, with poor management practices and where the primary purpose is meat production [24].

In Peru, several recent characterization or typification studies were developed, mainly in the Andean areas, where three farming systems were defined in Ayacucho (home consumption, self-consumption with the marketing of surplus products, and the sale of cheese and kids to local markets), and in the coastal valleys, where three distinct groups were categorized according to their management practices, production, and marketing purpose [9,12]. Only two studies were carried out in regions of the dry forest. One was developed over thirty years ago, where seven goat production systems were established in the Piura region according to their environmental, economic, and sociocultural aspects [19]. Meanwhile, Temoche [25] classified goat farming in the Piura region through sociocultural, productive, economic, environmental, and ecologic components and their relation to the capacity for adaptation and the perception of climate change. The main issues of goat production systems were management practices, forage availability (dependent on the precipitation in the arid zone), and the farmers’ economic resources.

Although these studies provide crucial information on how the goat production systems work in some areas of the dry forest, further updated data are necessary to understand goat farming in terms of socioeconomic, technological, and productive aspects. Moreover, a better diagnosis and analysis of different regions within the dry forest are required to compare whether the same factors are decisive or specific for all these areas. Therefore, the present study aimed to characterize and typify goat production systems in the most representative areas of the dry forest of Peru; to identify gaps and develop targeted strategies that enhance productivity, promote sustainability, and strengthen the livelihoods of producers in this vulnerable ecosystem; and to contribute to establishing development policies in the livestock sector.

## 2. Materials and Methods

### 2.1. Study Area

**Figure 1:**
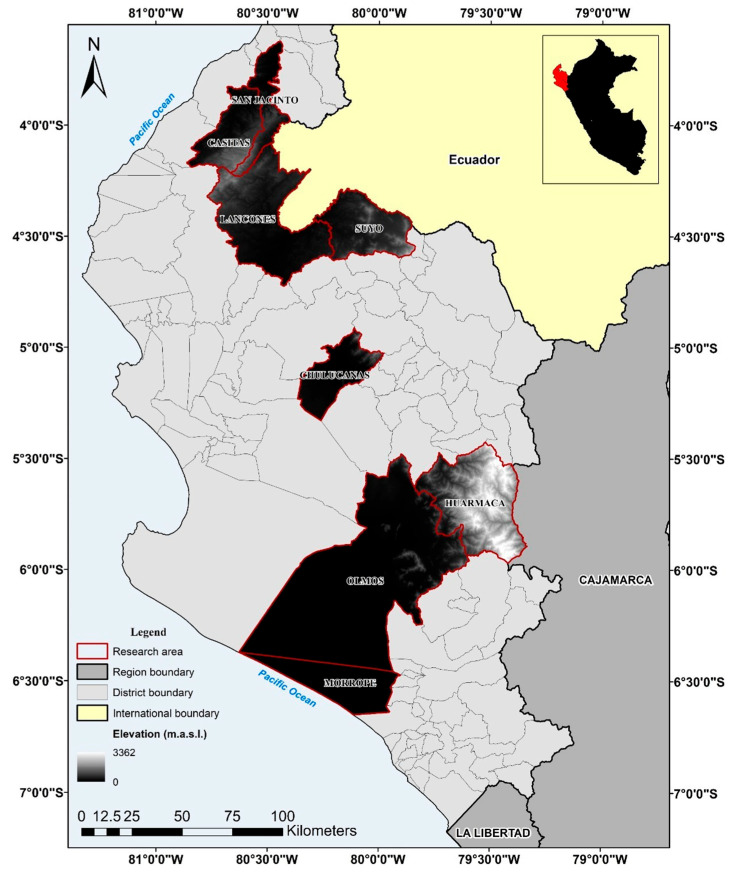
Map of the study areas in the dry forest of northern Peru.

The research was carried out in the regions of Tumbes, Piura, and Lambayeque, located along the northern coast of Peru within the tropical dry forest ecoregion. This specific ecosystem is defined by an arid climate, with annual temperatures being between 24 and 27 degrees Celsius and average annual precipitation rates varying from 100 to 500 mm. The area experiences a prolonged dry season, with a brief rainy period generally occurring from January to March [26]. Data were collected from populated centers within the dry forest across each region, offering a representative perspective of the ecosystem’s conditions and production systems, as illustrated in Figure 1.

### 2.2. Selection Criteria and Data Collection

Research sites were selected based on the predominant goat production systems in the tropical dry forests of northern Peru, covering the regions of Piura, Tumbes, and Lambayeque. To ensure adequate representation, 40 populated centers were identified to reflect the diversity of these systems’ sociocultural, productive, and economic conditions.

The selection criteria focused on sociocultural diversity (levels of education, organizational participation, and experience in goat production), productive variability (agricultural practices, technology usage, and livestock management), and economic diversity (market integration, income sources, and access to credit). These aspects are fundamental for characterizing the dynamics of goat production systems in arid regions.

A stratified sampling methodology was utilized to ensure the representative nature of the sample. The study encompassed 284 goat producers, who were selected based on their engagement in goat production and willingness to participate. Information was obtained through semi-structured, face-to-face interviews conducted with each producer. These interviews lasted approximately 30 min per visit and were designed to collect declarative data on production practices, socioeconomic factors, and environmental considerations. The gathered data were systematically organized into a database for subsequent descriptive and multivariate statistical analyses.

### 2.3. Selection of Variables and Statistical Analyses

The variables selected for this study were identified based on their significance in delineating the sociocultural, productive, and economic dimensions of goat production, following the methodology proposed by Pardo et al. [27]. A total of 48 variables were evaluated, seven of which were quantitative and the remaining qualitative. Table 1 clarifies the goals of characterizing and typifying goat production systems based on sociocultural, productive, and economic factors. The complete list of variable names, codes, and categories is available in the Supplementary Materials (File S1).

Multivariate techniques were employed to simplify the data structure and ensure robust interpretation. A principal component analysis (PCA) was used to identify patterns among quantitative variables. The Kaiser–Meyer–Olkin (KMO) test was applied to validate the data’s suitability for the PCA. Bartlett’s sphericity test was conducted to evaluate whether the correlation matrix was appropriate for the factor analysis.

Additionally, a multiple correspondence analysis (MCA) was applied to the qualitative variables, facilitating the identification of key dimensions. The reliability of the MCA was assessed using Cronbach’s alpha, ensuring that the extracted dimensions were internally consistent.

A factor analysis of mixed data (FAMD) was also conducted to integrate the qualitative and quantitative variables into a unified structure. This approach provided a more comprehensive understanding of interactions among the analyzed dimensions. Finally, a hierarchical cluster analysis (HCA) was performed using Euclidean distances and the Ward method to group the production systems into homogeneous clusters. The Ward method minimizes the total variance within each cluster and is particularly effective in producing clusters of similar sizes. This comprehensive methodology allowed for identifying distinct typologies within goat production systems. All statistical analyses were conducted using R software (version 4.3.3), ensuring precise data processing and visualization.

## 3. Results

### 3.1. Multiple Correspondence Analysis

The MCA allowed for a reduction in the initial 41 variables to the 15 most representative ones, selected based on their R^2^ (closer to 1) and *p*-value (*p*-value < 0.05). These variables were distributed into two main dimensions, explaining 22.07% of the total variability. Moreover, the Cronbach’s alpha values for the identified dimensions (dimension 1: α = 0.949; dimension 2: α = 0.925) reflected high internal consistency and ensured the reliability of the analysis performed (see Table 2). The complete list of the initial variables and their categories is available in the Supplementary Materials (File S1).

Dimension 1, which explained 13.63% of the total variance, is closely associated with infrastructure and technological factors within goat production systems. Among the most significant variables, the region (DEP) was the primary factor (R^2^ = 0.962, *p*-value < 0.01), highlighting marked differences between Tumbes, Piura, and Lambayeque in terms of access to resources and implemented production practices. The irrigation system (SRIE) also showed a significant contribution (R^2^ = 0.841, *p*-value < 0.01), reflecting how the use of gravity irrigation acts as a key indicator of modernization and higher productivity.

On the other hand, the problematic plant species variable (SPPROM) (R^2^ = 0.813, *p*-value < 0.01) revealed challenges associated with toxic plants such as *Ipomoea carnea*, especially in less technological systems. The goat farming reason ratio (DED) (R^2^ = 0.683, *p*-value < 0.01) showed that more technological systems tend to focus on commercialization, while traditional systems prioritize self-consumption. Furthermore, access to training (CAP) (R^2^ = 0.433, *p*-value < 0.01) reflected the positive influence of education on the adoption of advanced technologies. In contrast, the goat rearing area (ARCRIA) (R^2^ = 0.517, *p*-value < 0.01) highlighted that systems with larger territorial areas are better positioned to integrate innovation and diversification into their processes.

Moreover, it was observed that systems without structured animal identification processes (IDENT) (R^2^ = 0.452, *p*-value < 0.01) exhibit lower organization and efficiency levels in management, while the person most involved in livestock activities (TDES) (R^2^ = 0.498, *p*-value < 0.01) indicated that in traditional systems this responsibility mainly falls on the head of the household. The available infrastructure (INSTA) (R^2^ = 0.493, *p*-value < 0.01) emphasized the prevalence of basic structures such as pens in less technological systems, and the origin of sires (REEMP) (R^2^ = 0.389, *p*-value < 0.01) highlighted the importance of regional exchange as a source of genetic variability. Finally, corrective actions in affected areas with a problematic plant species control plan (ACONTR) (R^2^ = 0.397, *p*-value < 0.01) reflected active practices such as area cleaning.

Dimension 2, which explained 8.44% of the total variance, captured the sociocultural and economic aspects of the production systems. As in dimension 1, the region (DEP) (R^2^ = 0.748, *p*-value < 0.01) had a significant weight, reflecting how geographic differences influence social organization and production practices. The type of product obtained (PRODQYL) (R^2^ = 0.698, *p*-value < 0.01) helped differentiate market-oriented systems, such as those producing live kids, from systems focused on meat or self-consumption.

In this dimension, problematic plant species (SPPROM) (R^2^ = 0.539, *p*-value < 0.01) remained relevant, demonstrating the challenges faced by less organized systems. The reproductive efficiency was represented by the number of goat kids per birth (CRIASPART) (R^2^ = 0.563, *p*-value < 0.01). In contrast, the frequency of the deworming variable (TDESP) (R^2^ = 0.498, *p*-value < 0.01) reflected more frequent sanitary practices in technological systems. The lack of structured animal identification processes (IDENT) (R^2^ = 0.376, *p*-value < 0.01) again highlighted lower organization in the traditional systems. In contrast, the production objective (OBJP) (R^2^ = 0.449, *p*-value < 0.01) helped distinguish diversified and market-oriented systems from those focused solely on subsistence.

**Figure 2:**
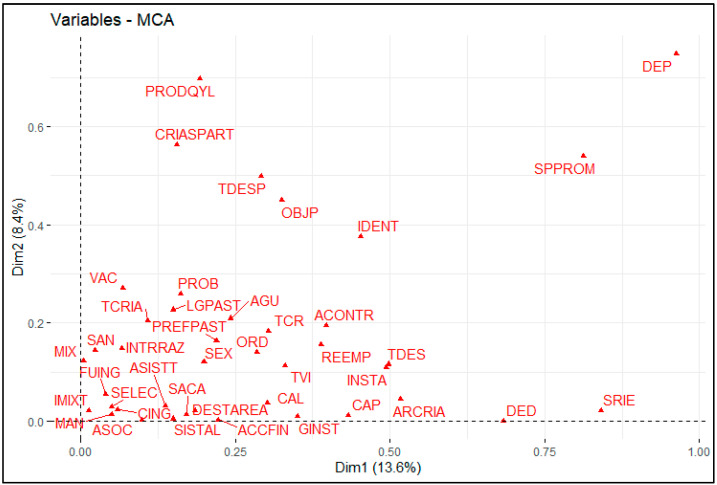
Graphical representation of the distribution of modalities for the first (Dim 1) and second (Dim 2) dimensions of the MCA applied to the typology of goat production systems.

Figure 2 illustrates the relationship between the selected qualitative variables in the two main dimensions identified by the MCA. This graph visualizes how the variables are distributed and contribute to the variability explained by each dimension, highlighting key associations within the goat production systems of the dry forest in northern Peru.

### 3.2. Principal Component Analysis

**Figure 3:**
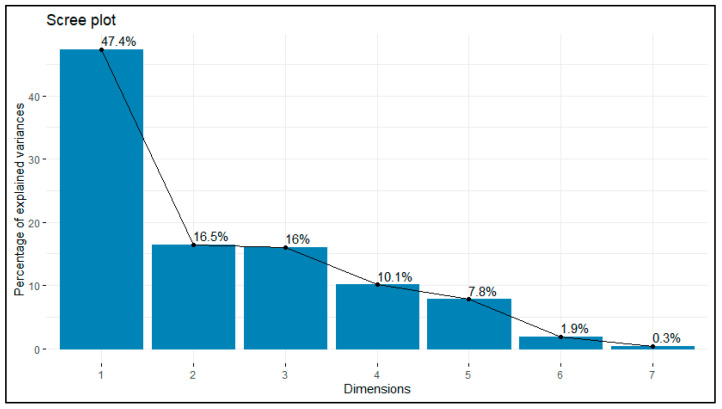
Percentage contribution of each principal component analysis dimension to the total variance.

The PCA allowed for identifying the fundamental dimensions explaining the variability in goat production systems. According to the scree plot (Figure 3), the first two dimensions explained 63.9% of the total variance, supporting their representative capacity in characterizing these systems. Dimension 1 explained 47.4% of the total variance and is associated with aspects related to population structure and productivity. In contrast, dimension 2, contributing 16.5%, is primarily linked to species diversification and population dynamics within the production systems.

To validate the suitability of the data for the PCA, the KMO sample adequacy measure and Bartlett’s test of sphericity were assessed. The KMO value obtained was 0.719, indicating an acceptable level of sample adequacy. Additionally, Bartlett’s test yielded a chi-square statistic of 1131.392, with 21 degrees of freedom and a significance level of <0.01, confirming the existence of significant correlations between the variables and validating the appropriateness of the data for the analysis.

**Figure 4:**
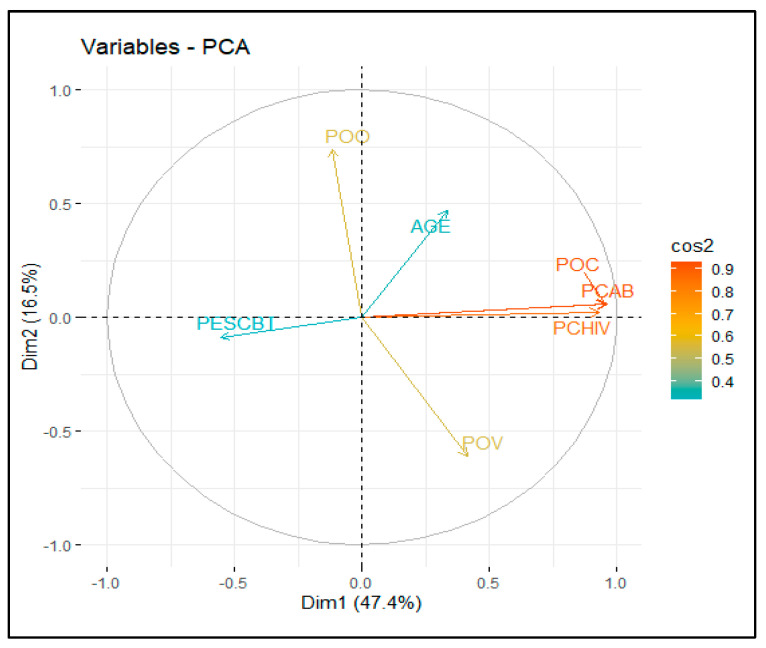
A graphical representation illustrates various dimensions’ contributions to the principal component analysis.

In dimension 1, key quantitative variables reflecting the goat systems’ population structure and productivity were identified (Figure 4). These included the total goat population (POC) (R^2^ = 0.958, *p* < 0.01), the adult female goat number (PCAB) (R^2^ = 0.962, *p* < 0.01), and the adult male goat number (PCHIV) (R^2^ = 0.931, *p* < 0.01), all showing a high correlation with this dimension. On the other hand, the average goat kid weaning weight (PESCBT) (R^2^ = −0.554, *p* < 0.01) exhibited a negative correlation with this dimension. In contrast, the average producer age (AGE) (R^2^ = 0.337, *p* < 0.05) also contributed significantly, reflecting its influence on the system’s structure.

In dimension 2, variables associated with species diversification and population dynamics were identified (Figure 4). The sheep herd size (POO) (R^2^ = 0.735, *p* < 0.01) was the most representative variable, while the cattle herd size (POV) (R^2^ = −0.613, *p* < 0.01) showed a negative correlation, suggesting lower integration with goats. Finally, the average age of the producer (AGE) (R^2^ = 0.470, *p* < 0.01) emerged as a relevant variable in the structure and management of the production systems.

### 3.3. Factor Analysis of Mixed Data

The FAMD identified significant patterns in the goat production systems of the dry forest in northern Peru, integrating qualitative and quantitative variables previously selected through the PCA and MCA. The initial reduction in variables resulted in 21 variables, of which 15 were obtained from the MCA and six from the PCA. To evaluate the contributions of these reduced variables, specific metrics were used, namely R-square for continuous variables derived from the PCA and a one-way ANOVA for categorical variables derived from the MCA. This approach explained 51.12% of the total variance across the first five dimensions, emphasizing the first two, accounting for 32.36% of the accumulated variability.

Dimension 1, which explained 21.3% of the accumulated variability, reflects a socioeconomic and agricultural practices gradient differentiating intensive and extensive goat production systems in the dry forest of northern Peru. Among the most significant continuous variables, the adult female goat number (PCAB) showed a positive correlation of 0.83 (*p*-value < 0.001), highlighting that systems with larger goat populations are associated with intensive production strategies and a high management capacity. Similarly, the total goat herd size (POC), with a correlation of 0.80 (*p*-value < 0.001), underscores the relevance of reproductive efficiency in these systems. In contrast, the adult male goat number (PCHIV), with a correlation rate of 0.76 (*p*-value < 0.001), supports the importance of reproductive parameters as a fundamental axis of differentiation. Conversely, the cattle herd size (POV), with a moderate correlation of 0.54 (*p*-value < 0.0003), indicates that integrating other species can complement agricultural management strategies in more developed systems. In contrast, the average goat kid weaning weight (PESCBT), with a negative correlation rate of −0.51 (*p* = 0.0007), suggests that systems with lower body weight animals tend to align more with extensive practices and lower levels of technology.

The ANOVA for the categorical variables highlighted that the geographic region (DEP) is one of the most influential factors, with an R^2^ of 0.95 (*p*-value < 0.001), whereby Tumbes is associated with intensive production systems. At the same time, Piura and Lambayeque reflect more diversified strategies. The goat farming reason (DED) showed an R^2^ of 0.90 (*p*-value < 0.001), evidencing that family tradition is a key driver in configuring productive systems. Similarly, the irrigation system (SRIE), with an R^2^ of 0.77 (*p*-value < 0.001), indicates that systems with access to gravity irrigation achieve higher productivity levels. Finally, the impact of problematic plant species (SPPROM), such as *Ipomoea carnea*, was demonstrated with an R^2^ of 0.72 (*p*-value < 0.001), emphasizing the need for control strategies in sustainable systems.

Dimension 2, which explained 11.1% of the accumulated variability in the FAMD, is mainly associated with the production orientation, sanitary management, and specific objectives of goat production systems. Among the continuous variables evaluated using R-square, the average goat kid weaning weight (PESCBT) showed a positive correlation of 0.51 (*p* = 0.0009). This suggests that systems with which goat kids attain higher average weights are associated with more organized productive strategies.

For the categorical variables evaluated through the one-way ANOVA, the results emphasized that the geographic region (DEP) had a significant impact, with an R^2^ of 0.93 (*p*-value < 0.001). Specifically, Piura stood out for being associated with diversified systems that integrate a higher frequency of deworming (TDESP = quarterly, *p*-value < 0.001). This sanitary management process, represented by an R^2^ of 0.63 for TDESP, reflects the relevance of parasite control practices for optimized animal performance and system sustainability. In contrast, the systems with annual deworming showed negative estimates (*p*-value < 0.02), indicating a possible adverse impact on the growth and development of kids.

The goat farming products (PRODQYL), with an R^2^ of 0.63 (*p*-value < 0.001), also proved relevant in this dimension. The systems focused on selling live kids achieved high estimates (*p*-value < 0.001), emphasizing their importance as a commercial strategy in these regions. Meanwhile, the production objective (OBJP) showed an R^2^ of 0.55 (*p*-value < 0.001), with the systems combining the production of live kids and milk.

Among the additional categorical variables, the management of problematic plant species (SPPROM), such as *Ipomoea carnea*, presented an R^2^ of 0.73 (*p*-value < 0.001), highlighting the need for effective control to avoid negative impacts on productivity. Specifically, the category SPPROM = purslane (*Portulaca oleracea*) showed negative estimates (*p*-value < 0.001), underscoring its adverse influence on goat systems.

Concerning the categories of regional variables, DEP = Piura was associated with dynamic and diverse systems. Conversely, DEP = Lambayeque displayed negative estimates (*p*-value < 0.001), suggesting less intensive systems or those characterized by infrequent management practices.

### 3.4. Hierarchical Cluster Analysis

**Figure 5:**
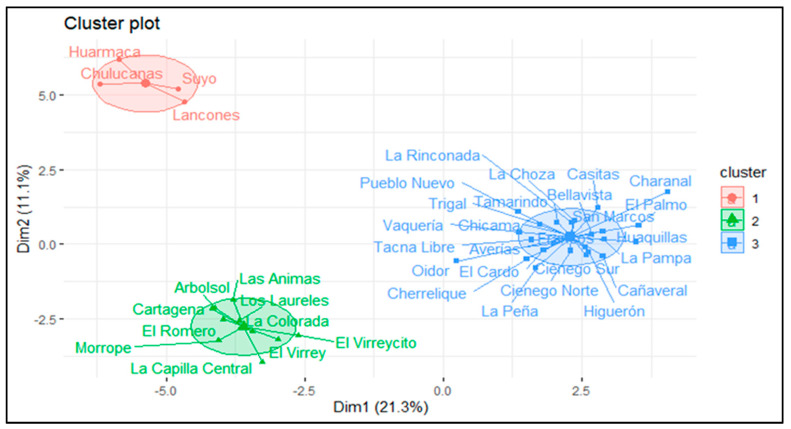
Distribution of goat production system clusters in the dry forest of northern Peru.

The cluster analysis identified three homogeneous groups of goat production systems in the dry forest of northern Peru (Figure 5). Each cluster represents different levels of intensification, productive management, and adaptation to the specific conditions of the dry forest in their respective regions.

#### 3.4.1. Cluster 1: Intensive and Commercial Production Systems

Cluster 1 (*n* = 96) includes goat production systems located exclusively in the Tumbes region (100%, *p*-value < 0.001). These systems exhibit high intensification, focusing predominantly on live kid production (100%, *p*-value < 0.001), reflecting a highly commercial and specialized approach. Regarding sanitary management, 80% of the systems implement quarterly deworming (*p*-value < 0.001), indicating a high level of preventive control. Additionally, 57.14% of producers clean affected areas as a sanitary management measure to prevent the spread of diseases (*p*-value < 0.001). Reproductive practices are characterized by a predominance of single births in 57.14% of the systems (*p*-value < 0.001), a strategy that minimizes risks associated with multiple births. Regarding grazing areas, 21.05% of the systems manage areas measuring between 0.5 and 1 hectare (*p*-value < 0.05), which is appropriate for the needs of this intensive management type.

Regarding productive objectives, 37.50% of the systems combine live kid and milk production (*p*-value < 0.01), demonstrating moderate diversification to complement their commercial focus. Moreover, 100% of the producers correctly identify toxic or problematic plants, such as *Ipomoea carnea* and other species (*p*-value < 0.01), a fundamental action to avoid poisoning risks and economic losses in controlled grazing systems.

Animal identification is implemented in 66.67% of the systems, facilitating herd control and traceability (*p*-value < 0.001). The motivation for engaging in this activity is primarily linked to economic and cultural factors, with family tradition and profitability being the main reasons in 26.67% of cases (*p*-value < 0.05). Furthermore, the decisions related to the management of the productive system are predominantly made by the family head (father) in 93.30% of cases (*p*-value < 0.05), reflecting a management model led by the paternal figure in the households of this cluster.

#### 3.4.2. Cluster 2: Extensive Systems Limited by Water Resources

Cluster 2 (*n* = 100) includes goat production systems located exclusively in the Lambayeque region (100%, *p*-value < 0.001). These systems lack controlled water resources, with 76.92% not using irrigation (*p*-value < 0.001), a condition associated with prolonged droughts in the region. Additionally, the producers face significant challenges due to the presence of toxic plants, such as purslane (*Portulaca oleracea* L.) and bitterweed (*Parthenium hysterophorus*), affecting 100% of the systems (*p*-value < 0.001).

Regarding productive management practices, these systems primarily focus on meat production (69.23%, *p*-value < 0.001). Replacement males are sourced from the herd in 52.63% of cases (*p*-value < 0.001), reflecting a traditional and self-sufficient approach to genetic management. The grazing areas are moderate, with 52.63% managing plots measuring between 0.5 and 1 hectare (*p*-value < 0.001). Single facilities for housing animals are used in 47.61% of the systems (*p*-value < 0.001).

The reproductive practices in these systems include single and twin births in 41.67% of cases (*p*-value < 0.01), reflecting a reproductive strategy adapted to environmental limitations. The decision-making regarding system management is shared equally between fathers and mothers in 40% of systems (*p*-value < 0.05), highlighting a family-centered approach to decision-making.

#### 3.4.3. Cluster 3: Technical Systems with Irrigation and Intensive Management

Cluster 3 (*n* = 88) consists of goat production systems found solely in the Piura region (100%, *p*-value < 0.001). Most of these systems utilize traditional irrigation techniques, with gravity being the most prevalent (96.15%, *p*-value < 0.001). These systems encounter challenges common to dry forests, such as the presence of toxic plants including jimsonweed (*Ipomoea carnea*), Overal (*Cordia lutea*), and creeper (*Ipomoea crassifolia*), impacting 92.59% of the systems (*p*-value < 0.001).

Regarding the management practices, the most used animal identification method is ear notching (80.65%, *p*-value < 0.001), a traditional system for identifying animal ownership in communal areas where goats practice controlled grazing. All systems use shared facilities for all goat categories and pens for nursing kids (*p*-value < 0.01), separating kids from their mothers during grazing periods. The grazing areas are predominantly smaller than 0.5 hectares (100%, *p*-value < 0.01).

The sanitary management practices include semi-annual deworming in 78.26% of the systems (*p*-value < 0.05). Additionally, 32% of the systems practice breeder exchanges with producers from other regions to improve the herd genetics. Regarding the production orientation, 30.77% of the systems focus on meat production (*p*-value < 0.01).

## 4. Discussion

### 4.1. Multiple Correspondence Analysis

The MCA was instrumental in identifying the most relevant qualitative variables that structure goat production systems in the dry forest of northern Peru. This approach reduced the initial 41 variables to 15 and captured patterns associated with geographic, technological, and socioeconomic factors differentiating these systems. The findings are consistent with previous studies employing multivariate methods to characterize complex systems in agricultural contexts, particularly in arid and semi-arid regions [5,12,28].

The number of selected variables and percentage of variance explained by the principal dimensions are key indicators of the MCA’s effectiveness. For instance, Palomino et al. [12] applied an MCA in goat systems in arid regions, selecting 18 variables from an initial set of 50 and explaining 28.5% of the variability across three dimensions. In comparison, this study reduced the initial set to 15 variables, explaining 22.07% in two dimensions. This study reflects the specific characteristics of northern Peru’s goat systems influenced by their unique ecological and socioeconomic conditions.

Similarly, Miranda-de-la-Lama et al. [5] analyzed small ruminant systems in Mexico using an MCA, reducing the variable set to 20, with 30% of the variance explained across three dimensions. This highlights how data heterogeneity can influence an MCA’s capacity to synthesize information—a phenomenon also reported by Akounda [28], who observed that high productive diversity contexts yield lower explained variance rates.

Like previous investigations, this study’s results emphasize the importance of combining an MCA with other multivariate tools to integrate qualitative and quantitative data. The internal consistency of the identified dimensions, measured through Cronbach’s alpha, supports the robustness of the results. Miranda-de-la-Lama et al. [5] also reported high internal consistency values (>0.90) in their dimensions, reinforcing the reliability of this approach.

#### 4.1.1. Dimension 1: Infrastructure and Technological Advancement

The first dimension explained 13.63% of the variance, capturing infrastructure and technological advancement factors. Differences between Tumbes, Piura, and Lambayeque were evident in aspects such as access to water resources and management practices. Studies highlight the importance of irrigation access in production systems in arid regions [29,30]. In this context, gravity-fed irrigation emerged as a marker of technological advancement, underscoring how water infrastructure availability leads to enhanced system organization [10,31] and efficiency [5,32].

Challenges associated with toxic species such as *Ipomoea carnea* reflect specific limitations in less technologically advanced systems. Barboza et al. [26] emphasized that the presence of such plants directly affects animal health and productivity. Additionally, technical training and basic infrastructure, such as pens for lactating kids and designated rearing areas, played a significant role in this dimension, aligning with recommendations by Palomino et al. [12], Rodríguez-Vargas et al. [33], and Morales-Jerrett et al. [6].

#### 4.1.2. Dimension 2: Sociocultural and Economic Aspects

The second dimension explained 8.44% of the variance, concentrating on sociocultural and economic factors. The market orientation, evident in producing live goat kids and derived products such as milk and meat, served as a key differentiator between technologically advanced systems and those focused on subsistence [28]. Research that involves sociocultural and economic aspects shows that productive diversification is a vital strategy for mitigating financial risks in vulnerable regions [32,34].

More frequent sanitary practices in technologically advanced systems, such as deworming, reflected greater adoption of advanced technologies [10]. However, the lack of structured flock identification remains challenging for traditional systems, limiting their access to specialized markets [5,30]. Furthermore, the presence of toxic species and limited resource management underscore these systems’ vulnerability to adverse conditions, as observed in studies conducted in semi-arid regions of Africa [35].

The MCA results provide a solid framework for understanding the differences among goat production systems and designing targeted strategies to improve their sustainability [28]. Implementing efficient irrigation and productive diversification practices in technologically advanced systems can enhance their market competitiveness [31]. For traditional systems, improving flock organization practices, controlling toxic species, and accessing basic infrastructure are priorities, aligning with proposals by Pateiro [34] and Martínez et al. [32].

### 4.2. Principal Component Analysis

The PCA identified two fundamental dimensions jointly explaining 63.9% of the variability in goat production systems. This level of explained variability highlights the PCA’s capacity to simplify the complexity of the original data, a finding consistent with previous studies in agricultural systems, where the effective variability representation rates typically range between 60% and 70% [33,35].

The acceptable level of sample adequacy (KMO = 0.719) and significant results of Bartlett’s sphericity test confirm the data’s suitability for the PCA [32]. These metrics align with previous studies validating the PCA for analyzing agricultural systems in similar contexts [12], where the KMO value was 0.730. Additionally, the scree plots and factor loadings obtained in this study are comparable to those reported by Hart [29], who found that the first two dimensions explained 65% of the variance in integrated small ruminant systems.

#### 4.2.1. Dimension 1: Goat Population Structure and Productivity

The first dimension, explaining 47.4% of the total variability, was strongly associated with population structure and productivity factors. Quantitative variables such as the total goat herd size (POC), the adult female goat number (PCAB), and the adult male goat number (PCHIV) showed high positive correlations with this dimension, reflecting the direct influence of the herd size and composition on productive dynamics. These results align with those reported by Martínez et al. [32] and Sarria et al. [10], who identified herd size as a determinant factor for profitability and sustainability in goat systems.

Conversely, the average goat kid weaning weight (PESCBT) showed a negative correlation, potentially indicating that larger goat populations face nutritional and reproductive management challenges. Studies have noted that higher animal density rates may limit access to food resources, affecting key indicators such as the weaning weight [5,12].

The producer’s average age (AGE) contribution to this dimension highlights the influence of accumulated experience and knowledge on managing production systems. Rodríguez-Vargas et al. [33] and Hart [29] emphasize that older producers tend to maintain traditional practices, which impacts productivity and the adoption of technological innovations.

#### 4.2.2. Dimension 2: Herd Diversification and Population Dynamics

The second dimension, explaining 16.5% of the total variability, was primarily linked to species diversification and system population dynamics. Variables such as the sheep herd size (POO) showed a strong positive correlation, reflecting the integration of complementary species in some goat systems. This diversified approach has been noted to mitigate risks and improve production systems’ resilience to environmental and economic fluctuations [11,26].

In contrast, the negative correlation of the cattle herd size (POV) suggests that these animals may compete directly with goats for limited resources, a phenomenon also reported by Martínez et al. [32] in extensive systems in semi-arid regions. Once again, the producer’s average age (AGE) emerged as a relevant factor, reflecting its impact on diversification and management decisions [35].

The analysis underscores the importance of optimizing the population structure and promoting species diversification for improved goat system sustainability. Nutritional management strategies are recommended in high-density systems to ensure optimal goat kid growth [3,6]. Additionally, integrating species such as sheep can complement production activities, provide additional income sources, and strengthen the system’s resilience to environmental and economic risks.

### 4.3. Factor Analysis of Mixed Data

The FAMD allowed for the integration of qualitative and quantitative variables selected through the PCA and MCA, thereby characterizing the goat systems in the dry forest of northern Peru. This approach explained 51.12% of the total variability across the first five dimensions, emphasizing the first two, which accounted for 32.36% of the cumulative variability. Studies such as those by Palomino et al. [12] and Miranda-de-la-Lama et al. [5] highlight that the variability explained in factorial analyses typically ranges between 50% and 70%, depending on the heterogeneity of the data. In this study, the 51.12% explained by the FAMD is comparable to reports from goat systems in semi-arid regions, reinforcing the methodological validity. Additionally, integrating qualitative and quantitative data through an FAMD provides a more detailed characterization [29,32].

#### 4.3.1. Dimension 1: Social Background and Agricultural Practices

Dimension 1, which explained 21.3% of the variability, highlighted significant differences between the intensive and extensive systems. Continuous variables such as the adult female goat number (PCAB) and goat herd size (POC) showed positive correlations associated with intensive systems with higher levels of technological management. This finding aligns with studies by Palomino et al. [12] and Gómez-Urbiola [11], which identified herd size as a key factor in goat productivity in arid regions. Similarly, Hart [29] reported that intensive systems require high efficiency in herd management to maximize production.

The use of irrigation, specifically gravity irrigation (SRIE), emerged as a marker of modernization, consistent with studies emphasizing that access to water is a determining factor in the sustainability of goat systems in semi-arid regions [3,5]. Conversely, challenges such as toxic species (*Ipomoea carnea*, *Portulaca oleracea* L.) limit productivity, a problem widely documented in pastoral systems [26,33].

#### 4.3.2. Dimension 2: Farming Production and Sanitary Management

Dimension 2, which explained 11.1% of the variability, reflected the importance of sanitary practices and productive objectives in differentiating systems. The deworming frequency (TDESP) was particularly relevant, showing that more technologically advanced systems implement more frequent management practices. This is consistent with studies linking regular sanitary control to higher animal productivity [3,25,32].

The goat farming products (PRODQYL), such as live kids or milk, underscore diversification as a key strategy in market-oriented systems [35]. These findings align with research indicating that productive diversification enhances producers’ incomes and strengthens resilience to economic and environmental fluctuations [5,36].

One of the most relevant implications of these findings is the potential to develop new markets focused on younger consumers. Alcalde et al. [36] emphasize that considering regional consumption preferences and habits, specific commercial strategies can be designed to boost the demand for goat products. This is particularly important for systems with basic infrastructure and diversified productive practices, enabling better market integration and more significant economic benefits for small-scale producers [37].

The FAMD results highlight the need for differentiated interventions. For intensive systems, it is recommended that access to advanced technologies be strengthened and resources, such as water, be managed sustainably. For extensive systems, policies should prioritize the control of toxic species and technical training to improve the resilience of productive systems, as suggested by Palomino et al. [12] and Laouadi et al. [37].

### 4.4. Hierarchical Cluster Analysis

The cluster analysis identified three homogeneous groups of goat production systems in the dry forest of northern Peru, highlighting diversity in production management, the levels of technological advancement, and adaptation to the agroecological conditions of the region. This type of analysis has proven to be a key tool for characterizing agricultural systems, as noted by Palomino et al. [12] and Gispert-Muñoz et al. [2], who identified clusters differentiated by levels of technological advancement and access to infrastructure in goat systems from arid regions. Similarly, Paredes et al. [9] reported a similar segmentation in goat systems in the Peruvian coastal region.

Moreover, Oliveira et al. [21] and Laouadi et al. [35] emphasized the utility of multivariate approaches in classifying production systems in semi-arid contexts and identifying clusters based on the degree of agricultural integration, management strategies, and production objectives. This study’s findings are consistent with the previous research and underscore the relevance of multivariate analyses for understanding the heterogeneity of goat production systems, enabling tailored interventions for the specific needs of each group.

#### 4.4.1. Cluster 1: Intensive and Commercial Farming Systems

Cluster 1 comprises highly technologically advanced systems in Tumbes, focusing on live goat kid production and advanced sanitary management. These findings are consistent with studies highlighting the link between the commercial orientation and the implementation of advanced practices, such as quarterly deworming [33], reported in 80% of the analyzed systems. Additionally, cleaning affected areas as a sanitary management measure (57.14%) reflects effective disease mitigation strategies, as Laouadi et al. [35] reported in intensive small ruminant systems.

The use of animal identification methods (66.67%) and product diversification toward milk and live kids (37.5%) align with prior studies emphasizing the importance of these practices for improving traceability and competitiveness in technologically advanced systems [12,26]. Furthermore, the effective control of toxic plants such as *Ipomoea carnea* was reported as essential for ensuring the sustainability of intensive systems [38].

In this context, Dubeuf et al. [38] proposed reevaluating the relationship between natural resources and productive activities through agroecological models. This is particularly important in cluster 1, where intensification must balance resource valuation with improved goat productivity [21]. This approach not only maximizes productivity but also promotes sustainability [37], reinforcing the strategic role of these systems in local food security and economic development within the dry forest [39].

#### 4.4.2. Cluster 2: Extensive Systems Limited by Water Resources

Cluster 2, located in Lambayeque, is marked by dependence on limited natural resources and extensive practices, reflecting structural challenges in production sustainability. The absence of irrigation systems in 76.92% of the cases aligns with studies highlighting that a lack of water infrastructure significantly reduces productivity in arid regions [32,39]. Additionally, the presence of toxic species such as *Parthenium hysterophorus* and *Portulaca oleracea* L. in 100% of the systems affects sustainability, a phenomenon also documented by Melo et al. [40] and Gómez-Urbiola [11].

A notable aspect of this cluster is that 40% of decisions related to livestock management and household activities are made jointly by both parents. This finding is supported by Rodríguez-Vargas et al. [33] and Thu et al. [41], who emphasize that in low-input rural systems, responsibilities are shared between heads of households as a strategy to optimize the management of limited resources and strengthen decision-making in contexts of high economic vulnerability.

This shared decision-making pattern underscores the importance of equitable participation within the family unit in improving the efficiency of extensive systems [42]. This factor should be considered when designing intervention programs for sustainability and resilience in goat systems [37].

Finally, as noted by Escarreño [23] and Morales Jerrett et al. [6], transitioning to more sustainable and productive strategies requires a systemic approach that integrates animal nutrition, health, genetic improvement, technical knowledge, and accessible technologies [21]. This approach fosters practices that contribute to the sustainability and resilience of goat systems in resource-constrained environments [7,30].

#### 4.4.3. Cluster 3: Production Systems with Irrigation and Intensive Management

Cluster 3, located in Piura, is characterized by advanced productive strategies, particularly gravity irrigation (96.15%), a practice widely associated with increased productivity in technologically advanced systems. These findings align with those reported by Miranda-de-la-Lama et al. [5] and Schneider [37], who highlighted that access to water is a determining factor for sustainability in semi-arid regions. Moreover, semi-annual deworming (78.26%) reflects an intermediate level of technological advancement, comparable to Hart et al. [29], who observed that adopting sanitary technologies significantly improves animal health and system efficiency.

However, as Oliveira et al. [21] note, producers in this group also face significant challenges in managing limited resources and modernizing technological infrastructure. These challenges and the need for more efficient management practices emphasize the importance of incorporating innovative technological tools. Sharma et al. [43] propose that integrating monitoring and management technologies can optimize productive decisions and promote more resilient and sustainable systems.

Furthermore, Dubeuf et al. and Salgado et al. [1,38] argue that developing agroecological production models that value local resources and sustainably manage inputs is key to strengthening the competitiveness and sustainability of these systems. Therefore, combining technological strategies, adequate infrastructure, and agroecological practices could transform the systems in this cluster into modern and resilient production models in the face of climate change and economic fluctuations.

### 4.5. Limitations and Future Research

This study provides significant insights into goat production systems in the dry forest of northern Peru; however, it presents some limitations that should be considered for future analyses. An inherent limitation is its cross-sectional design, which while allowing for the identification of significant patterns through multivariate analyses and cluster identification does not capture the temporal dynamics that may influence the evolution of production systems, such as seasonal changes in resource availability or the cumulative impacts of adverse climatic factors.

Additionally, using self-reported data through interviews may have introduced biases, particularly in income, productivity, and technology adoption variables. Although cross-validation was implemented to mitigate this bias, the subjective nature of some data may limit the precision of the findings. Furthermore, excluding more detailed environmental variables, such as soil quality or biodiversity impacts, restricts the ability to take a comprehensive view of the studied systems.

Based on these limitations, the future research directions include longitudinal studies to capture temporal dynamics, detailed analyses of climate change effects on system resilience, and an economic approach to evaluate the feasibility of technological interventions. It is also suggested that sociocultural factors be investigated using qualitative methodologies, goat product diversification as a financial strategy be considered, and the impact of monitoring and management technologies for real-time practice optimization be assessed.

## 5. Conclusions

This study provides a detailed characterization of goat production systems in the dry forest of northern Peru, highlighting heterogeneity in production management, the levels of technological advancement, and the adaptations to the agroecological conditions of each region. Using multivariate analysis tools, three clusters were identified, representing distinct production dynamics. Cluster 1, comprising intensive and commercial systems in Tumbes, stands out for its technologically advanced management approach focused on live goat kid production and derived products, demonstrating high productivity and market orientation. Cluster 2, encompassing extensive systems in Lambayeque, faces challenges such as a lack of water infrastructure and toxic species but underscores the importance of family-focused decision-making. In the case of cluster 3, located in Piura, the predominant use of gravity irrigation and adoption of advanced technologies were observed, although limitations related to infrastructure modernization and communal management persist.

These findings emphasize the need to implement differentiated public policies that address the specific characteristics of each cluster. Promoting investments in basic and technological infrastructure is imperative, particularly in extensive systems lacking adequate water resources. Additionally, technical training programs are essential for efficient resource management and to encourage the use of innovative technologies, especially in systems transitioning toward technological advancement. Product diversification should also be encouraged by creating specialized markets for products such as live kids and dairy derivatives, facilitating producers’ integration into more competitive value chains. Moreover, it is crucial to integrate sustainable and agroecological practices, including effectively controlling toxic species and strengthening local capacities for sustainable resource management.

Another key aspect is recognizing goat ecosystems and cultural services. Valuing and compensating for these services would ensure their sustainability and contribute to the socioeconomic development of rural communities. Finally, it is essential to articulate these initiatives within an integrated approach, where governmental, local, and private actors work together to reduce production gaps, improve producers’ livelihoods, and ensure resilience to the challenges posed by climate change.

## Figures and Tables

**Table 1:** Objectives of each component within the characterization process.

Sociocultural	Production System	Economic
A. Housing	A. Farming system (intensive or extensive)	A. Farming production (meat, milk, subproducts)
B. Social background	B. Agricultural practices	B. Income
C. Educational attainment		C. Credit
D. Projections and challenges		
E. Participation in organizations		

**Table 2:** Statistical summary of the main dimensions from the multiple correspondence analysis.

Indicator	Dimension 1	Dimension 2
Percentage of explained variance	13.63	8.44
Eigenvalue	0.279	0.173
Cronbach’s alpha	0.949	0.925
Number of selected variables	11	7
Cumulative contribution (%)	13.63	22.07
Total inertia	0.312	0.237
Most representative categories	DEP, SRIE, SPPROM, DED, CAP, ARCRIA, IDENT, TDES, INSTA, REEMP, ACONTR	DEP, PRODQYL, SPPROM, CRIASPART, TDESP, IDENT, OBJ

## Data Availability

The data presented in the current study are available on request from the corresponding author.

## Supplementary Materials

The complete list of the initial variables and their categories is available in the supplementary materials (File S1). The following supporting information can be downloaded at https://www.mdpi.com/article/10.3390/ani15040567/s1. Supplementary File S1. List of variable names, codes, and categories used in the surveys.
