# Supplementary material for: Characterization of Goat Production Systems in the Northern Dry Forest of Peru Using a Multivariate Analysis
Source: Animals (Basel). 2025 Feb 16;15(4):567. doi: 10.3390/ani15040567 (PMC11851541; doi:10.3390/ani15040567)
Supplement: Supplementary file 1 [file animals-15-00567-s001.zip › animals-3376315-supplementary.pdf]

Nº	VARIABLE	Código	Modalidades
1	Rural Settlement	SEC	CP1-CP40
2	Region	DEP	Tumbes, Piura, Lambayeque
3	Age	AGE	Quantitative
4	Sex	SEX	Male, Female
5	Type Person	PERS	Natural person, Legal Entity
6	Level of education	GINST	No education, Elementary education, High School Education, Higher education
7	The person with the Greatest Involvement in Livestock Activities	TDES	Wife, Husband, Both Spouses, Entire Family
8	Time engaged in goat production	TCR	< 5 years, 5 – 10 years, 10 – 20 years, >20 years
9	Housing Characteristics	TVI	Mud with Wooden Frame (adobe), wood, concrete, Cane and Mud (quincha), Other
10	Economic Income in USD	CING	$0–132.28 USD, $132.54–264.55 USD, $264.81–529.10 USD, >$529.10 USD
11	Main Source of Economic Income	FUING	Agriculture, Commerce, Livestock farming, Agriculture and Livestock, All sources
12	Main Problematic Area	PROB	Social, Environmental, Commercial
13	Associativity	ASOC	Yes_ASOC, No_ASOC
14	Training	CAP	Yes_CAP, No_CAP
15	Technical Assistance	ASISTT	Yes_ASISTT, No_ASISTT
16	Reason for Dedication to Goat Farming	DED	Hobby_1, Profitability_2, Main regional activity_3, Family tradition_4, Low investment_5
17	Production System	SISTP	Intensive, Extensive, Mixed
18	Production Objective	OBJP	Meat production_1, Milk production_2, Livestock sales (live animals) _3, Self-consumption_4, Dual-purpose_5
19	Hours dedicated to goat farming per day	TCRIA	Less than 3 hours, Between 3 and 5 hours, - Between 5 and 6 hours, - Between 6 and 9 hours, More than 9 hours
20	Destiny of Agricultural Area	DESTAREA	Agricultural, Agricultural and cultivated pastures, Forests and shrubs, cultivated pastures, Natural pastures, Natural pastures combined with forests and shrubs.
21	Irrigation System Used	SRIE	No irrigation, Gravity irrigation, and technified irrigation.
22	Area Dedicated to Goat Rearing	ARCRIA	less than 0.5 hectares, between 0.5 and 1 hectare, between 1 and 2

			hectares, and more than 2 hectares, None
23	Mixed Rearing with Other Species	MIX	Yes, Not
24	Goat Population	POC	Quantitative
25	Sheep Population	POO	Quantitative
26	Cattle Population	POV	Quantitative
27	Does Population	PCAB	Quantitative
28	Buck Population	PCHIV	Quantitative
29	Types of Facilities Used	INSTA	Differentiated corrals, unique corrals, unique corrals equipped with water troughs; unique corrals with shelters for resting, and pasture areas,
30	Mixed facilities	IMIXT	Yes, not
31	Feeding practices for goat farming	SISTAL	Native pasture, native pasture with supplementary with balanced feed, native pasture combined with agricultural residues
32	Ownership of grazing areas	LGPAST	Private area, communal area
33	Water source for livestock	AGU	Canal, river, stream, drinking trough in the pen
34	Problematic plant species for goats	SPPROM	*Ipomoea carnea, Ipomoea, portulaca oleracea crassifolia, Prosopis spp.*, , Other species
35	Problematic species control plan	ACONTR	Avoiding the area and treating the affected animal, Preventing grazing in those areas, Taking no control measures, Taking no control measures but treating the affected animal, Cleaning affected areas, Cleaning affected areas and treating the affected animal, Treating the affected animal.
36	Preferred forage species	PREFPAST	*Medicago sativa*, *Prosopis spp., Zea mays, Sorghum vulgare, Acacia macracantha, Capparis scabrida,* Other species
37	Identification system used	IDENT	Ear notches, tattoos, ear tags, none.
38	Management Practices	MAN	Yes, No
39	Crossbreeding within the herd	INTRRAZ	Yes, No
40	Type of reproductive practice used to achieve pregnancy in a female goat.	SERV	Natural mating, controlled mating, artificial insemination
41	Origin of breeding males used as sires, sourced from the producer's herd, regional suppliers, or external regions.	REEMP	From own herd, from other regions, from the region
42	Criteria used by the producer for selecting replacement animals	SELEC	Individual performance, parental performance, visual evaluation, others
43	Number of kids per birth	CRIASPART	Single, double, triple, single and double, all types

44	Percentage of goat offspring selected by the producer for sale	SACA	Yes, No
45	Milking practices	ORD	Yes, No
46	Health management	SAN	Yes, No
47	Health calendar	CAL	Yes, No
48	Indicates whether the producer performs regular deworming in their goat herd.	DESP	Yes, No
49	Refers to the frequency or timing of deworming practices implemented by the producer.	TDESP	Semiannual, Quarterly, Annual, no deworming, Only when the animal is sick
50	type of products derived from goat farming	PRODQYL	Milk, Cheese, Does not sell, Manure, Meat, Live kids, All
51	Access to financing	ACCFIN	Yes, No
